# Supplementary material for: Identification and Fine Mapping of a Locus Related to Leaf Up-Curling Trait (Bnuc3) in Brassica napus
Source: Int J Mol Sci. 2021 Oct 28;22(21):11693. doi: 10.3390/ijms222111693 (PMC8583815; doi:10.3390/ijms222111693)
Supplement: Supplementary file 1 [file ijms-22-11693-s001.zip › Table S1.pdf]

Table S1. The designed primers of SSR and InDel markers used in this study.

Name of Marker	Types	Forward primers(5'-3')	Reverse primers(5'-3')
BnA02V0001	SSR	ACCGTAACTTGTAACCTTTT	CAGTATGTTTAGGCTGTGGG
BnA02V0007	SSR	ACGCCTATAAAATCGGTAC	AATCAAATGCTTTCCTTGT
BnA02V0011	SSR	ATACCATATTTCTCCACCAG	CTCTTTGATTTCACCTTTCT
BnA02V0013	SSR	CAACGGCGGCGAAGAGGAT	TACGGAGGCAGTGGTGGTGG
BnA02V0014	SSR	CAACAACTTCTTGCCTTATCT	TTACGCTGGTACGTCATTC
BnA02V0017	SSR	GGTCGGAACTGGACACTAT	CTGCCTGTAACAAGAAAGC
BnA02V0018	SSR	GGCGTCTTAAATATCAAAGT	CAATCAAACCGAATAAATGG
BnA02V0020	SSR	CTAACAAAACCGTAGATGACC	TGCCTTGAACTGTACAATAAA
BnA02V0028	SSR	TTTTCCTACTTCAATTACAG	ATGATTTACTAGTCGGGTTA
BnA02V0030	SSR	CCTGCCTGGTTAGATTTGACG	CGCCGCACCGAATAAGAAGAA
BnA02V0035	SSR	AGTTACAATAAATCTCCTCATC	TATCGTATACACCTAGCAAAG
BnA02V0039	SSR	GCGACGAAATCTCCTACACCT	GAAATGGAAATTGAAACCCTGA
BnA02V0045	SSR	GTCTGACCCCTGGTGTTTTGG	ACACTTCTCCTTACACTGTGCTTGA
BnA02V0053	SSR	GCCCATGACCGCCCTGAATAT	TCCGTGAGGCTCCAACTGAAT
BnA02V0056	SSR	ACCGATTCAGGAACTCGTC	AGAGTGGGAAGTCCTATGC
BnA02V0059	SSR	AGGGTAGGAATAACTGAAT	ACTTTACTTTTAATGTGGG
BnA02V0064	SSR	GAATAACCACCGACACCAA	AGAAGGGAGGCAAAGCATA
BnA02V0068	SSR	TCACAATCAACATCCCAATA	TTTATGCCGACTTCTCAAT
BnA02V0072	SSR	AGCTTTGGTTTGTTTGATTT	GTGGTGGTGATTGTGATGAT
BnA02V0076	SSR	GGTGACCACTACGGAGACAA	CATTGCTGCGAGAAGGAAGA
BnA02V0079	SSR	GAACAATATCAAAGGGCAAG	GCAGCAGTCAGAAGTAGAAG
BnA02V0082	SSR	GAAAAGGATGTGGGTGGAGA	CTGTGAGCGACGGAGAAGAG
BnA02V0095	SSR	CAAACTAACCGACTCTGACG	AACAATGGAGGGAAACAATA
BnA02V0099	SSR	CTCCGTGATTTCATTCTCAA	CTCATCGTCATATCCAGTGTT
BnA02V0103	SSR	GATAAGACCCGTGCCTTG	CTGATCAATTTAGTCGTCCAGT
BnA02V0105	SSR	GTTCTTGAAAATTCATTGAAAG	CGACAATAAATGACTAAAACTATT
BnA02V0112	SSR	TTTGGTGATGAATAATGTAAC	GTACAGTTCTTGAGGAACACT
BnA02V0123	SSR	CGATTGATTCAGATTCTTATTG	ACTTTTCATCTTTACCACCAC
BnA02V0126	SSR	TTGTTTCTCATGGGTTAGT	CTCATCACAAAAGGCTTAG
BnA02V0127	SSR	GTTTGTTTCTCATGGGTTAG	AGCCAGCTTCAACATTACTA
BnA02V0133	SSR	AGATTAGACATGGGGAAGCTG	CCATAACCTAAAAGGATACCA
BnA02V0140	SSR	CCAGCTTCACTTTCTTAAT	CAGCAGTCTTTGCTTCTAG
BnA02V0146	SSR	GGTAATAGCGTTCCTTGACA	CGGAACTTCTACTCTCTGGC
BnA02V0149	SSR	GAGAATTAAACTCGGAAGTCG	AAAGTGTTTCTAAACCTCAAA
BnA02V0150	SSR	TAACCATCTCTCACCATTTG	TGATTCTTTGATCGTCACTT
BnA02V0152	SSR	CTATTCCTAAGCCTACTCAAATC	TGGCATATCATCTGTGTGC
BnA02V0154	SSR	AGTGTTTGTGTTCGTGTGAC	CCTCCCTCTGATGTCCAC
BnA02V0156	SSR	AAAGTATGGTTTGGTTCAGTT	TAAACTGTTTAACATTTGCAC
BnA02V0158	SSR	TTGGTCTCACCTTTTATGTCT	CTTATCAAAAATCATCATAACAAT
BnA02V0160	SSR	CAAAAATAGCACAAAGGAGA	TCACTGAATCATAAACCCAC
BnA02V0161	SSR	CTCCTCCACCCGAGATACTA	TAAGTTGGTGTTGGTGGCTA
BnA02V0162	SSR	CAACAACAACAAGAGAAAGA	TCACCAGAACAACTGAATAA

BnA02V0165	SSR	CATCAATACATCAGCCACTATG	TTGGAATGAAATGAGAAAGA
BnA02V0166	SSR	CTTGTCCTATGGATTTTCAGT	CCAAAACACACGTAACCTTG
BnA02V0174	SSR	TTTGGGTAAGAATCTTGGTT	TACCGATTCAAACAGTCATACA
BnA02V0175	SSR	ATTGTATGACTGTTTGAATCGG	ACTTATCAAGAAAGCAGAAGTTAC
BnA02V0176	SSR	CACCAAGGATAAACTCAAGGGAT	CATCAGGCTAAGAATGTTCATACAA
BnA02V0178	SSR	AAATGAAAAGTAATCCGTGTT	TCATTAAACAGATTCACGAAA
BnA02V0186	SSR	TCTCATTTGAACCAATCGCA	GCCGTAAAGTTGGGAGAGGA
BnA02V0188	SSR	CAACAAGGCAATCAAACG	GGTGGCTCGTGGTAGTTC
BnA02V0189	SSR	CCCATAGATTCCCTGGAT	ATTAATCAAAATTCATTGCAC
BnA02V0190	SSR	TCTTGGGCTGAGATGTAAAT	TTGGGTTTATATCAAACATGA
BnA02V0191	SSR	TAGGTTAACAATTCACATTT	AATTAGTAACTTTCATCCCA
BnA02V0193	SSR	AAAAGTCCTAGACAAATAACCAAA	AGCTGTCTTAACGACGGAGA
BnA02V0194	SSR	GAACATAGGAGCCTAACGAA	CCGATTTGTACCCACTCATA
BnA02V0195	SSR	GGTAATGGGGAAAAGTGG	TCGGACAAAACGAATCAG
BnA02V0197	SSR	ACATAGGAGCCTAACGAAGG	CAAAACGAATCAGATCACCA
BnA02V0198	SSR	TTCAATAAATAAAATACTGTAAAAC	TCCTCTACGCTCATTCTAAC
BnA02V0199	SSR	GTCAGGGACGTAGGGTACAC	ATCACCTTAAATCCAGTTGG
BnA02V0200	SSR	AGAAGCCAAACCTCAAATAAG	GACGCAGTTTCAGCCATTA
BnA02V0201	SSR	GAGGGAGAACAATTTAGTAGAG	AAGTGAAGTCAAAACAAGGAA
BnA02V0202	SSR	AGGTGTAGGAATGTGTAGAAGT	TTCAAAAATTATTATAACTGTGC
BnA02V0203	SSR	AAATATCGAATTTGAAAAATTA	CTTTACCACCACTAAAAGAACT
BnA02V0206	SSR	GGCTATGCCATCTTCTTTTGT	GTTATCAGACTTGTGAACCGT
BnA02V0207	SSR	TGAAGAAACCCTCTGACAACA	GGTCATCCGAAGACTTAGAAA
BnA02V0209	SSR	CTGAAAAGAACGTCAACATCT	AATCACTCAAACCCATTAGGA
BnA02V0213	SSR	CGATTACACCAAAATAGGTCA	CTTGCTTCTATTCATTTTCATTC
BnA02V0216	SSR	TGCTAAACTCCGCAACAA	GCAGAGGCTAAGGAAACG
BnA02V0217	SSR	CCAAAGGTGTAGGGAGATAGA	CCTGGGCCTAATTTCTTGTAG
BnA02V0220	SSR	ACACCTACCTATCTAAGCAAC	GATGTCTATAAATGAACAACG
BnA02V0221	SSR	GACTTGAAACTTTTAGAGGTGT	TACTACATACTTGGAGGAAATAA
BnA02V0222	SSR	AGACGCAGTGAACTCTTTAG	AGTATACCGAGGCTTTGGTA
BnA02V0223	SSR	ACGGTCCAGTCCGTCTCT	ACCTTGTTGATAAATGATAAGTT
BnA02V0224	SSR	CCGAGACTGAGCATCTAAACT	GGGAAGCAATAAAACTAAAACAA
BnA02V0225	SSR	CAAATATGATAGAGAATGCACTAAT	ATCACCACAATGAATTTATGG
BnA02V0226	SSR	ACCTCAAATGTTGTAATGATTCT	GTACTCCGACTACAATACACTTT
BnA02V0227	SSR	AAAGGACCCAAGCAACATTCT	TAGGCTCAACGACAACCAAAA
BnA02V0228	SSR	ATAGGCTCAACGACAACCAAA	AAAGGACCCAAGCAACATTCT
BnA02V0232	SSR	CATGAAACCATCCAAAACCTT	GGCTATGGGTGAACATTGTCT
BnA02V0234	SSR	TCGGTTTCATCACCCTAA	TTGTCCTCTTCCATTTCG
BnA02V0236	SSR	AAGAAGAGGAAGGCTTAAACA	CACAAGACAATAGGCGTGAGA
BnA02V0237	SSR	CAAATGCTACTGACTTGAAAG	GACAGAAGGAACAAAGAAATC
BnA02V0240	SSR	TTTGTTTTCTGATGGTGGACT	GTCATTTCAGATAATTGTAAACGAT
BnA02V0242	SSR	TATACTAAATCGCACATGCTC	TTGATTTCTTCATTTCTGGAT
BnA02V0245	SSR	ACAATAAATTTTGGATAAGGAG	TTAGGAACAGAGGGAGTATAA
BnA02V0246	SSR	ACAATAAATTTTGGATAAGGAG	TTATTTAGGAACAGAGGGAGT

BnA02V0251	SSR	TCGTTCCTCCGCTAATGTTCT	CCTCCTCATCTTCTCCTCCCT
BnA02V0254	SSR	TATTATGGAACGGAGGGAGTA	GAACCATCAACTATACCACCT
BnA02V0260	SSR	GCCAACAATACCTCTGAATAA	AGTTTAGCGAGAAAATGTGAC
BnA02V0262	SSR	CAAACATAACAATTGTAGCGTT	GTCTTGATATACCAGGAAGGAG
BnA02V0263	SSR	AAACCTTTGAGAACTCCCTGAT	CCACTCTTGATGTTCTGTCCC
BnA02V0266	SSR	TAAACCTTCAAAACTCCAATC	TACATAAGCGAAAGAAACAGA
BnA02V0269	SSR	AAGACGGTGACGAATTGAGGG	TGTTCACGAAATCTCCAGGTA
BnA02V0279	SSR	ATGGATATTTGTTTCATTTGAG	TTTTATGTTGGTGTTGTAGCC
BnA02V0283	SSR	CATGTTGGAAAATCAATGGTA	GAAATAGTTAAAAGTTAGGTGG
BnA02V0287	SSR	AAATCATACAGTCACCGAAACC	TGCCATAGGACAACGAAATAG
BnA02V0289	SSR	GTAACAGCAACAGAAAGCCC	CTCTAATGAAAACCAACGCA
BnA02V0291	SSR	GCGTGTCTGGAGATACATAAC	TGATAAGAATAGCCCTAGGAT
BnA02V0293	SSR	TTTTGGACTTCAGAATAACTT	TCTGGAGATACATAACTTGGT
BnA02V0302	SSR	CAGATCGCCGAAGACTAGAAC	AAACGAGGCAAGTGAAATGTA
BnA02V0308	SSR	ATTCGAGAAACCCAACCAGAG	AACGGAGATTTGATCCCAACG
BnA02V0314	SSR	CTTCTATTTGTTGTTGTTATG	GAGTTTTGTGAAAGTTTAGTC
BnA02V0318	SSR	CCAAAATGGTTCATCGAAGAG	TAAACCTCAATCCTCAAACCC
BnA02V0320	SSR	TGAACAACTAAAACAAATTG	AAAAGCCTCAAAAGAAAA
BnA02V0323	SSR	TTCCAAATCAATCCCATC	TCTCGTTACAAGCCACAG
BnA02V0331	SSR	ACATCATAGTTCTAAATTCCAA	GGTTTATCCACAAAGTCACAT
BnA02V0333	SSR	CGTTGAAAAGTTATAGTGATTATGC	AAGTTTATGGTTACGGGTGGA
BnA02V0336	SSR	ACGAATACAATAACAAATCTC	TTTTGATGCGTCTTACTTATA
BnA02V0342	SSR	AAGTATTTATTTTGAGGTATGC	GTAGATTTCTCCACTATTTGA
BnA02V0353	SSR	GTCGGCTTATTTCATTCG	GGTTGTTGTTGCCTTCAC
BnA02V0365	SSR	ATGTAATCAGCCTCAAGTAAT	ACGGGATTATAGTAGGAGATA
BnA02V0367	SSR	CGACCGCCCACAGCTAAT	GCGGGTTTGACGGGTTAC
BnA02V0374	SSR	ATCTCCTAGTGCAAAGATAA	TGTTAAAGCAAACAACCAAT
BnA02V0390	SSR	TCATTTGACCTGCGATTTTA	AGACATGGCCGGGTTTCTTT
BnA02V0398	SSR	ATATCATTTCTACAAAACTC	GGTAGTAATTGTACCTAGTG
BnA02V0405	SSR	TATTTCGCAAACACTGTGAC	GTTCATTTTAATTCATGGTTC
BnA02V0407	SSR	CTCTAGGCATCCAGTTTTATA	TTTCACTCTCATCCATAATCC
BnA02V0410	SSR	TATTATCATCATCTTCACGCC	TACTTCAAATCTTCTGATAACAAA
BnA02V0422	SSR	TGATTTCTCTGTTCTGCTCCT	CTTCCCGTTTCAATATTCTCT
BnA02V0495	SSR	ATCATCCTTTCATAGCCATCT	CTCGGAGCATTATTGACTTTA
BnA02V0536	SSR	TGTCGTCGTCTTCGTTGG	AAGAAACCGTTCCAGAGG
BnA02V0539	SSR	CGCCTATCACACACACCT	CGAGGTGAAGTTGTGGAG
BnA02V0540	SSR	TCGCATTCCAATAACTTCA	TCTCCTTTCTTTCCCCTTT
BnA02V0542	SSR	CAAGTTACGAGAAAGGGGA	CGTTGAGTTGAGTCCCATT
BnA02V0548	SSR	TAATGTTCATTGATGATAAGCA	TTAGGGAAATTGTATCATCTATT
BnA02V0572	SSR	ACTCAAATAGCGATAGAAAT	TCTTCGGAATCTGAAGAG
BnA02V0573	SSR	ACAACAGCCAAACCAATCA	GGATAACGACGGTGAAGAT
BnA02V0576	SSR	CTGGCTGTAATCCACAATCTA	TTGGTAGATGCAAGAATCAAA
BnA02V0586	SSR	CGTATTAAGGTTTTAACGAGTTG	TTGGTTCCAGCGATTTGAT
BnA02V0588	SSR	CCCAACATAAACGGAAGAGT	GTCATAATATATAAACGCCATGTAG

BnA02V0595	SSR	CAAGCAGAAGCAAGAGTCATT	GACACCTGAAACGCTCAAATA
BnA02V0628	SSR	TAAACCAAACATTTATCCATATG	AATTGTCTTTTCATATTTCACTC
BnA02V0643	SSR	CTCCCGTCAATAGCCATC	CCTTTTCGAGTCGAACAA
BnA02V0673	SSR	CATAAGTTCTTGAGGTGGGAG	TGGTAAGGATGAGATTGGGTA
BnA02V0686	SSR	TTGAGTTGACTTGTTCGCTTA	AGAGTTTCACAAACAGCAATG
BnA02V0704	SSR	TCAACCTATCAATTAGCCAAAA	AGAAACTGGTTGCCAAAGAA
BnA02V0705	SSR	TGTGCTGATGAAATCCTTAGAG	AGTTGCTGAACGTCAACCTA
BnA02V0711	SSR	ATCTTTCTTCGTCTAGCTTC	CTCTTTAACTCATGGATAATAA
BnA02V0713	SSR	GATACGCTGGACTGGAACAA	ATCTAAACGTCAACGCACAA
BnA02V0714	SSR	AAAAGAAGTAAGAGGCGGGTGT	TCAAAGGAGTCGTGTAGAGGCT
BnA02V0717	SSR	ATTTGAAGGTAGACACTGAA	CTCTTGTCTTATCTGGCAGG
BnA02V0723	SSR	AACAGATAAGTGATTAAAAC	TCACTAACTATTTTCTTTTC
BnA02V0726	SSR	TCAAACTATACCAGGCAAGA	AAACAACATACGTTCAAAGC
BnA02V0734	SSR	TCCAAATAGGATTCTACATT	AGAAACATCTTTAGCCAGTG
BnA02V0737	SSR	ATCATTTACAAACACTCGCAA	CGATATGAGAATTTGCGTACA
BnA02V0738	SSR	ACAAACACTCGCAACTAATAAC	GGAAAGACAGGCAGCTAGATAA
BnA02V0740	SSR	AAAGACAGGCAGCTAGATAA	AAACACTCGCAACTAATAAC
BnA02V0742	SSR	TTGTGCTTGCTTTGCCGAGA	CTGTGGAGCTGTGAGGTATAGTGG
BnA02V0751	SSR	AAGCACCATTTCAACCTCAA	TAAGCAAGCCATTTAACCTA
BnA02V0759	SSR	AGGTCGAAACTTAAGCTACT	GAAGATACAAATACATGTGAAATT
BnA02V0765	SSR	TTGATTCAGTCTTTCTCAAAG	TTGAATAACATCAAACGGTATA
BnA02V0766	SSR	GTATGTCATGTATGAATGCA	TTTTAATGTTGGACCTCTAG
BnA02V0771	SSR	CCATACTATGTTAGAAAGTTTA	CATCAAGAACAATACCATAA
BnA02V0776	SSR	AAAGCAAAAGCAAAGACAAG	CAACGCAAAGCAAACATTAA
BnA02V0779	SSR	CAAAGTAGAAAACCGACAAA	TTAGGATAACATTGCTGAAT
BnA02V0781	SSR	CAAGATACAAATGATTACAA	TTCATAATTTACCTTCCTAA
BnA02V0783	SSR	TAGTATACCCCTCTATCATTTT	TTGTAATCATTTGTATCTTGTC
BnA02V0789	SSR	CCATTTTACATTATTTGAACGT	GTTATGCCAGAGTCAGGATTAT
BnA02V0796	SSR	TTTGTAGGGTCGGTATTCTT	TGTAGGTGGCGTTCCTGTCT
BnA02V0812	SSR	ATGGTTTGTGAGAAGATAGTC	CAGAAACGATGAGAGAAGTG
BnA02V0903	SSR	CTTCGTGGTTTGCTTTACT	AGCCTCCTGTTGATGATTT
BnA02V0906	SSR	TATACGGGACCAGGAGATG	TACAAATTCGAGCCAAAGC
BnA02V0915	SSR	TGATAAAGGAAACACTATGATG	ATGACGAGATATTTGGCTATAA
BnA02V0919	SSR	CCTTCATTCAATTCAAAACC	TGTTGTTTAGATTTGGGGAT
BnA02V0931	SSR	ACCACGAGTTTCCCGTTTCTTT	GCTAGATCCATCGCGTAGTTCA
BnA02V0934	SSR	GTTCGGTTACAGAATTTGCA	CTTTAGGGTTTTGGCTCGGT
BnA02V0942	SSR	AATCGGAAACGTCACTTACT	ATGGTCCTTTCTTACTCTTCTT
BnA02V0944	SSR	TGTGTCAAGTTTTGGAACATC	ACGTTGTAAATAGTTAATGAAATT
BnA02V0955	SSR	TGATGTGATTTGGTGTTAGT	GATGATTTTAATTTACAGCATG
BnA02V0958	SSR	CCCATACCCTCCGATAAGTT	AAGGGAATAAGATTCAGCAA
BnA02V0968	SSR	TAAGCCTATAATAAGGGAGA	GCTCTGTTTAATCAAAGGAA
BnA02V0969	SSR	TAAGCCTATAATAAGGGAGA	AAAGGAAATTGCTATGAAGA
BnA02V0992	SSR	ACATCGTTAAGGTTTTATTC	AAAGACTCATCAGGTGGTAG
BnA02V0999	SSR	ATTGTTGTTCCTCACATTGC	CTGCCTTATGAAGTTAATTTTATC

BnA02V1009	SSR	GGTTGTTCTATTTCGGTGTA	ATGTGGCTTTATCTTGTTGG
BnA02V1012	SSR	ACGTCGAATGAAAGTGGTGG	TTGGCGGAAGATGCTGTAAG
BnA02V1039	SSR	TTATAGGCAAATGGACCAAA	TCCCAAGTCTAAATGGCAAG
BnA02V1051	SSR	TCATTCTCCAAACAAACTCA	TTGATTCTCACAATCCCATA
BnA02V1060	SSR	GATTTGAAAATGCATGTGAT	TGTCCCTTCGTATGTTGTTA
BnA02V1073	SSR	TGAGCACCAACATTTCACTT	AAACAAGGAGGAAGAAGAAG
BnA02V1075	SSR	AAACAAAACAAATACCCACT	AACCAGGCTCTAATATCAAC
BnA02V1081	SSR	CGGAAACATATTTTACCTGAA	AACAAGTGAGAGAGGGAGAAG
BnA02V1086	SSR	ATGTGATCGGCTAGGGAGAA	ATGGTTGGGACTTGAGGAAA
BnA02V1089	SSR	TGCCTCCCACCATATTTCTA	AATAAGCCACGTCACCTCAA
BnA02V1138	SSR	AACATTGTGCTTCTTTTCTCA	AGTTACATCAGCAGCACAAAG
BnA02V0413	SSR	TGTATGACTGTTTGAATCGG	GTTTGAGAAACGAGATAACACA
BnA02V1336	SSR	TCTTATTCCCAATCTTCACTTG	TGTTTGATTTGGATTTGAAG
BnA02V1337	SSR	AAGTATTCAACTCACTGTAAGC	ATATTTTGGGATGATATTATGA
BnA02V1338	SSR	TCTATGAGCACAAAAGTTACGTT	CCAAACGAACAAATTCCG
BnA02V1339	SSR	TTTTGCACATTTGCTATATT	TAACTTGAACTTCTGGGAGA
BnA02V1340	SSR	GAAGTCCGCATAGATTATTT	AAGGCTTTTTACAGTCACAT
BnA02V1341	SSR	TTCTCTTAGCAAACTATCGTTG	ACTGAAAATCCATAGGACAAG
BnA02V1342	SSR	AAGTGTATATTTACCGCCTAAC	TTTCATGTCTCTGGTTTGAG
BnA02V1343	SSR	TCGTGAGTTTGCTTCCCTAT	ATTGCTCGTATCGTAAAAGG
BnA02V1344	SSR	TTGAGAAAGAAAACGAAGGT	AATTTACTTCTTTATCTTGAATTAG
BnA02V1345	SSR	TTTAGAAATTGTATGGCTTACT	CAACCAATCACTCCCATAA
BnA02V1346	SSR	GCTAATCCAAACCAGAAATA	ATTCTCTTCTTTTATCCCGT
BnA02V1347	SSR	CACATTTTATTTCACTTTCCA	ATTGCTTGCTTCTCTCAGCA
BnA02V1348	SSR	GGAACTAATCAATGTAGCAGAA	TTCTTATTTTACGAATCATTGT
BnA02V1349	SSR	AAAATATTCGATCTAACCCG	AATGCCACAAATCATGTCTA
BnA02V1350	SSR	ATAACATAAGTGGCTGGCTA	AATACGTCCCGTTTGAATAC
BnA02V1351	SSR	CAACTTTGAAACCTTTGAAC	TACAGTCCAGAGAATTAGCG
BnA02V1352	SSR	CCTTCCACATTGGTGTGATA	GCTCGTAGTGTTCTGGTTTT
BnA02V1353	SSR	TAAATCATAACTTCTTGGGC	ACATGAATTGTATATTACTGACAG
BnA02V1354	SSR	TTAGTGGTATGACTTCTCGG	ACTTTTCCCCATTACCTG
BnA02V1355	SSR	ATAAACACAATGGTCTTGGC	ACAACTTTTATGGTGTGTCG
BnA02V1356	SSR	CCTTACACAAGAGCGACCG	TGGATGTTTTAGAGTTGACCA
BnA02V1357	SSR	ATTAAGATTGTTAAAAGATTGACA	ATCTTCATTTTTCACTCTTCC
BnA02V1358	SSR	GTTTTAGGTTTGCATCTGCA	TCACAACATAACGAAGCCAC
BnA02V1359	SSR	AGTTATACAATGCGAAAATAGA	AATAAATTCAATGTCAAATCAA
BnA02V1360	SSR	AGGGGAGGAAATCAGTCAAC	CTTACAGCCTCAGGAAACG
BnA02V1361	SSR	ACGACGAACAAATATCTGGG	GAGGGGTGAGCCTTTCTCAA
BnA02V1362	SSR	AAACTAAAGCCCAAAACCCT	CACGTGGAAACTCACTCCCT
BnA02V1363	SSR	TTGTTTATGTCTATCTCGCA	TCGTCTGATTGGAAAGCA
BnA02V1364	SSR	GATACTAAGGGGCACAAACC	CCTCAACTTACAATCCTTTCC
BnA02V1365	SSR	GAAACCAAGAAGCAGTAGAA	GAAGACCGAATCAGAGAATA
BnA02V1366	SSR	AAGGAGAAGTGGGGTTTGTG	TAATTCCTTGTTGTCCCCAT
BnA02V1367	SSR	GACAAAACAGAAGAAACCCG	ATTGAATAACAAATCTCAATCG

BnA02V1368	SSR	TTTGCTATTTGTTTCCAACA	CAACCACAGACCATTTCATT
BnA02V1369	SSR	AGAAAGGAACGACTTACATTC	GTTTCATTATTCTTCAGGCA
BnA02V1370	SSR	AATCTTCATCACCATCCATC	GCTATTTCTATTTGCCTTGG
BnA02V1371	SSR	TAACTTGTGTACTTCTGCCTAT	ATGGTTCATTCTTGTTTGGT
BnA02V1372	SSR	AAACAAACCTTCCCAGCA	TGCTGAAGCCGAAGTATG
BnA02V1373	SSR	GATACGAAATGAACAAGAGG	CTCTTCTGCTATCCTCCTTC
BnA02V1374	SSR	ATGGACAAAACAGAAGAAACC	AACAAATCTCAATCGTTCGT
BnA02V1375	SSR	AGGAGTTACCAAAATGACG	TGTAGGTGGAGGAGAAGAGT
BnA02V2321	SSR	TCAATTAAAAATATTATATAGCTT	ATCGTGGCTGCTCTCATT
BnA02V2322	SSR	ACAAGCGGTATTTTGGTATC	AGCAAGAATGTTATTTCGTA
BnA02V2323	SSR	TAACATTCTTGCTTACAATTGTTC	GCTAAGTCTCAGGTAAGAATAA
BnA02V2324	SSR	GAAAGAAGCATGAGCCACATA	GCTCAGTAGGTACCATAACGC
BnA02V2325	SSR	TCAATTGTATAAGCCCTCCTG	ATGAGAACATGCTCAGTAG
BnA02V2326	SSR	GTTCAATTGTATAAGCCCTC	GATGACTCGGTACAGATTA
BnA02INDEL1	InDel	CGTGGTTGGAGGATTGGTG	CATCGATCTTCTGAACTAAAACAC
BnA02INDEL2	InDel	GTTTTACATTGGAGATGCTC	CAATATTCCACATTCCTCGT
BnA02INDEL3	InDel	CACTTATGATTTTGAAGTTACTTA	GAACTTCAATGATCAATGG
BnA02INDEL4	InDel	GATTTATTAATCAATTACAAAGA	GAACCGTATGATTATAAATTCTT
