# Supplementary material for: Identification and Fine Mapping of a Locus Related to Leaf Up-Curling Trait (Bnuc3) in Brassica napus
Source: Int J Mol Sci. 2021 Oct 28;22(21):11693. doi: 10.3390/ijms222111693 (PMC8583815; doi:10.3390/ijms222111693)
Supplement: Supplementary file 1 [file ijms-22-11693-s001.zip › Table S2.pdf]

Table S2 The designed primers of comparative sequencing used in this study.

Name of Primers	Sequence of primers	Product length
BnaA02T0156200ZS-F	CTAAGGGAAATAATCAAACGGA	1473
BnaA02T0156200ZS-R	GCCAGCTTCAACATTACTACCA	
BnaA02T0156300ZS-F	GGTCTGTCTTTATCTCCCAC	2258
BnaA02T0156300ZS-R	CACCAAATCAGTCTTCTCTT	
BnaA02T0156400ZS-F	AAACGGTGGTGCATCTTCG	1969
BnaA02T0156400ZS-R	GAACTAGACAATGTACCGCACACT	
BnaA02T0156500ZS-F	ACAATAAACCGATATAACTGTCT	1470
BnaA02T0156500ZS-R	AGGAAAGGATTGTAAGTTGAG	
BnaA02T0156600ZS-F-1	AATTCGCCGACAACGACTC	1359
BnaA02T0156600ZS-R-1	GGTGTCACAATCTACAGGACGG	
BnaA02T0156600ZS-F-2	CTTGTAACCGTTATGACGCA	1487
BnaA02T0156600ZS-R-2	ATCACAGACTCACAGTACCCAA	
BnaA02T0156600ZS-F-3	CTTGTAACCGTTATGACGCA	1270
BnaA02T0156600ZS-R-3	TTACAGGAGGAGCGGGATA	
BnaA02T0156600ZS-F-4	AGACTTCTGGACCTGGCTCAC	1143
BnaA02T0156200ZS-F	CTAAGGGAAATAATCAAACGGA	
BnaA02T0156700ZS-F-1	CGACGAACATTAAGCTAATTATC	1971
BnaA02T0156700ZS-R-1	CATAGCTGAAGGCAGAATTG	
BnaA02T0156700ZS-F-2	GACTGTTTCTATCCTCACGG	2239
BnaA02T0156700ZS-R-2	CAGATGGGAATGGAGTAATAG	
BnaA02T0156700ZS-F-3	CAGCCTACTCTTCGCTCTAATA	2491
BnaA02T0156700ZS-R-3	TGCTCCAAGATAACAACCC	
BnaA02T0156700ZS-F-4	CCTCGCTCCCAACTACATC	1942
BnaA02T0156700ZS-R-4	ACCAGGACCTCCTATAATGTAC	
BnaA02T0156800ZS-F-1	GGAGATTGTGTCGGAGATGC	1225
BnaA02T0156800ZS-R-1	CAACCAATAAAGCCAGCGT	
BnaA02T0156800ZS-F-2	GCTGGCTTTATTGGTTGC	1230
BnaA02T0156800ZS-R-2	AACACCTTTACTATCTCCATCA	
BnaA02T0156900ZS-F-1	TGTGGTGATGAGTTCACCGAC	1394
BnaA02T0156900ZS-R-1	ACATGTTCTGGTCTACTAAACGG	
BnaA02T0156900ZS-F-2	TCACTTAGAGCAGCTCCATTAG	1153
BnaA02T0156900ZS-R-2	TATTATCACCGTTCATTGCTC	
BnaA02T0157000ZS-F	GCTTATAGTTATCAAACCATAACG	667
BnaA02T0157000ZS-R	ACATACGACGAACATTCATTGA	

BnaA02T0157100ZS-F	TTCTGTTGATTTCAGCATTACT	1142
BnaA02T0157100ZS-R	CGTACAAACAACGTATCCG	
BnaA02T0157200ZS-F	CCAAACTAAAAAACCACAACGG	2140
BnaA02T0157200ZS-R	GCACAAAACCGCTTTATTCG	
