# Supplementary material for: Identification and Fine Mapping of a Locus Related to Leaf Up-Curling Trait (Bnuc3) in Brassica napus
Source: Int J Mol Sci. 2021 Oct 28;22(21):11693. doi: 10.3390/ijms222111693 (PMC8583815; doi:10.3390/ijms222111693)
Supplement: Supplementary file 1 [file ijms-22-11693-s001.zip › Table S3.pdf]

Table S3 The designed primers of quantitative RT-PCR used in this study.

Name of Primers	Sequence of primers
BnaA02T0157000ZS-qRT-F	AGAGTCATCGAGGCTGCAAG
BnaA02T0157000ZS-qRT-R	ACATGGTCCGCGTTTCTTCT
Actin7-qRT-F	TGAAGATCAAGGTGGTCGCA
Actin7-qRT-R	AGAAGGCAGAAACACTTAGAAG
